# Influence of ecological traits on spatio-temporal dynamics of an elasmobranch community in a heavily exploited basin

**DOI:** 10.1038/s41598-023-36038-y

**Published:** 2023-06-13

**Authors:** Federico Maioli, Benjamin Weigel, Elettra Chiarabelli, Chiara Manfredi, Alessandra Anibaldi, Igor Isailović, Nedo Vrgoč, Michele Casini

**Affiliations:** 1 Department of Biological, Geological, and Environmental Sciences, Laboratory of Marine Biology and Fisheries, University of Bologna, 61032 Fano, Italy; 2 Faculty of Biological and Environmental Sciences, Organismal and Evolutionary Biology Research Programme, Research Centre for Ecological Change, University of Helsinki, 00100 Helsinki, Finland; 3 EABX, INRAE, 33612 Cestas, France; 4 CoNISMa, 00196 Rome, Italy; 5 Department of Life Sciences, University of Trieste, 34127 Trieste, Italy; 6 Institute of Oceanography and Fisheries, 21000 Split, Croatia; 7 Department of Aquatic Resources, Institute of Marine Research, Swedish University of Agricultural Sciences, 45330 Lysekil, Sweden

**Keywords:** Ecology, Ecology

## Abstract

Elasmobranchs, which include sharks and batoids, play critical roles in maintaining the integrity and stability of marine food webs. However, these cartilaginous fish are among the most threatened vertebrate lineages due to their widespread depletion. Consequently, understanding dynamics and predicting changes of elasmobranch communities are major research topics in conservation ecology. Here, we leverage long-term catch data from a standardized bottom trawl survey conducted from 1996 to 2019, to evaluate the spatio-temporal dynamics of the elasmobranch community in the heavily exploited Adriatic Sea, where these fish have historically been depleted. We use joint species distribution modeling to quantify the responses of the species to environmental variation while also including important traits such as species age at first maturity, reproductive mode, trophic level, and phylogenetic information. We present spatio-temporal changes in the species community and associated modification of the trait composition, highlighting strong spatial and depth-mediated patterning. We observed an overall increase in the abundance of the dominant elasmobranch species, except for spurdog, which has shown a continued decline. However, our results showed that the present community displays lower age at first maturity and a smaller fraction of viviparous species compared to the earlier observed community due to changes in species’ relative abundance. The selected traits contributed considerably to explaining community patterns, suggesting that the integration of trait-based approaches in elasmobranch community analyses can aid efforts to conserve this important lineage of fish.

## Introduction

Elasmobranchs (hereafter also sharks and rays), which include sharks and batoids, play crucial roles in maintaining the integrity and stability of marine ecosystems, often being at, or near, the top of the food chain^1,2^. Sharks and rays grow relatively slowly, take many years to mature, and produce relatively few offspring compared to teleosts^3^. These characteristics make elasmobranchs inherently vulnerable to fishing^1,3,4^. Over the past 50 years, oceanic shark and ray populations have decreased by more than 70%^5^. Similar declines are also documented for coastal elasmobranchs^6–10^. It has been estimated that over one-third of all species are now threatened with extinction, mainly as a result of overfishing^11^. Habitat loss and degradation, climate change and pollution are also compounding factors of this decrease^11,12^. Despite the critical role that elasmobranchs play in regulating marine ecosystems, our current understanding of their biology and ecology remains incomplete. This is further complicated by the issue of unregulated, misidentified, unrecorded, aggregated, or discarded catches, which makes obtaining accurate, species-specific landing information challenging^13–15^. As a result, understanding the structure (i.e., the types and numbers of species present) and dynamics (how communities change over time and vary in space) of elasmobranch communities remains a major research topic in conservation ecology^12^.

The establishment and persistence of species in a particular location are the results of the combined effects of environmental filters (which correspond to those abiotic factors which prevent the establishment or persistence of species in local communities)^16^, biotic interactions^17^ and stochastic processes (e.g., dispersal^18^). In turn, the response of species to these factors is mediated by their traits^19^ and constrained by phylogenetic relationships^20^ such that for instance closely related species might display a similar subset of traits even though they may live in very different habitats. As a result of these processes, we observe variation in the number, abundance, composition, and traits of species present in different communities over time and space. Understanding the ecological processes underlying changes in natural communities has been challenging in part because of the lack of a coherent statistical framework enabling to infer actual assembly processes from community data. A promising framework for modeling community structures and dynamics is joint species distribution modeling^21^, which allows to simultaneously explore community-level patterns in how species respond to environmental gradients, estimate interaction across species and, more recently, link such patterns to species traits and phylogenies^22^. There are several advantages modeling multiple species jointly. One significant advantage is the ability to capture correlations in abundance among taxa and to use this information to improve estimates for rare species. This phenomenon is known as “borrowing strength” and occurs when more common species that share similar environmental responses with rare species are included in the model. Furthermore, incorporating species traits into the modeling process provides mechanistic insights into the processes that shape ecological communities and their dynamics^19,23^. Traits can be used to identify the drivers of species co-occurrence, interactions, and responses to environmental changes, leading to a more comprehensive understanding of the complex relationships between species and their environment^22^.

Accordingly, here we use such an approach to assess the demersal elasmobranch community of the North-Central Adriatic Sea, a heavily exploited basin^24^ where elasmobranchs have been depleted historically^25,26^. Elasmobranchs in this basin face the highest level of threat when compared to the other areas in the Mediterranean Sea. Recent assessments indicate that 70% of Adriatic species are regionally threatened, with 42 out of 59 species at risk^27^. Elasmobranch fisheries have been historically significant in this basin, and while a few species are still intentionally fished for food consumption, the majority of shark and ray catches are incidental captures in fisheries targeting more valuable teleost species^28^. Demersal elasmobranchs are primarily affected by interactions with bottom trawlers and trammel nets, although recent reports have also implicated pelagic and midwater trawlers in the northern Adriatic Sea^29^. Fisheries have developed at different rates in the eastern and western parts of this basin; high-capacity fishing fleets in Italian waters have put significant pressure on elasmobranch populations, while fishing exploitation in former Yugoslavian waters has been much lighter and only later expanded^30^. However in the last 20 years the fishing capacity decreased as effects of the decommissioning scheme adopted by the EU^31^. As a result, the number of trawl vessels has decreased by 36% between 2004 and 2015, with a similar decrease in Gross Tonnage and a reduction in days at sea by more than 50%^32^ (for an illustration of the recent trend in fishing effort, see Supplementary Fig. S1). Moreover, fisheries management plans have implemented various measures to protect the fish stocks, such as temporal and spatial fishing restrictions^33^.

Whereas several studies documented elasmobranch community depletion in the North-Central Adriatic Sea^7,34–36^ no attempts have been made to relate traits to community assembly, specifically examining to what extent spatiotemporal variation in occurrence and abundance patterns can be related to traits (but see Ferretti et al.^7^ for species intrinsic rebound potential, which is a measure of species ability to recover from exploitation). In fact, despite their comparative traits, shark and ray species exhibit fine differences in life-history traits^3,37^ and trophic level^38,39^ which likely influence the outcomes of biotic and abiotic filtering on species occurrence and abundance across space and time. Identifying the importance of these species’ ecological characteristics offers the potential to gain community-level insights as well as predict community dynamics. This knowledge can then be leveraged to develop targeted conservation strategies, such as implementing stricter fishing regulations or establishing protected areas and ultimately aid efforts for the conservation of this important lineage of fish.

**Figure 1:**
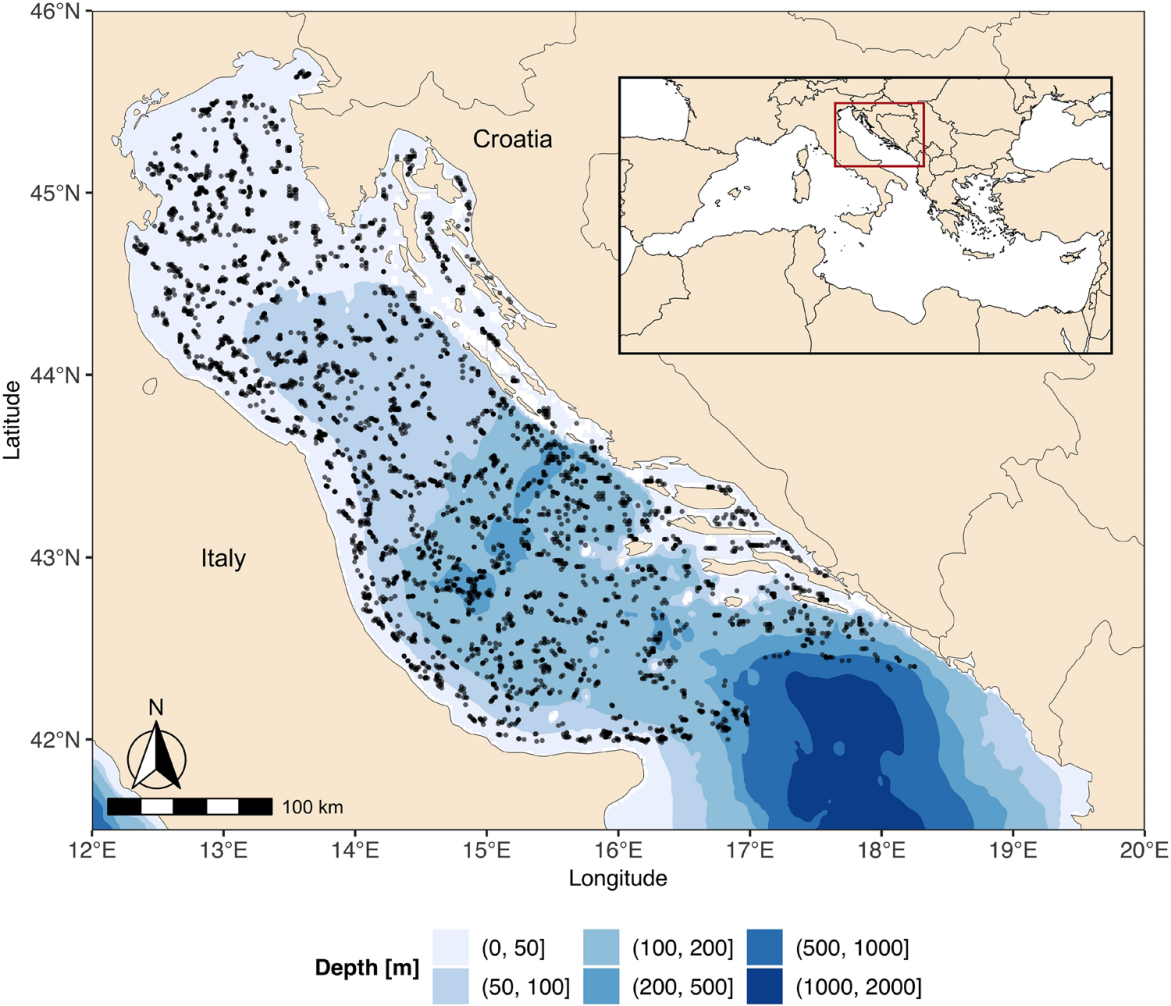
Map showing the location of the study area and the positions of all the analyzed MEDITS bottom trawl hauls for the period 1996–2019.

Here, we assess elasmobranch community changes in the North-Central Adriatic Sea by considering key life-history traits (i.e., age at fist maturity and reproductive mode), trophic level, phylogeny, and environmental factors. Specifically, we aimed at (a) understanding the spatiotemporal changes of the elasmobranch community in relation to environmental factors, (b) quantifying how much of the responses of the species to their environment can be ascribed to traits or explained by phylogenetic correlations. We employed Hierarchical Modeling of Species Communities (HMSC)^40,41^, a statistical framework that uses Bayesian inference to fit latent-variable joint species distribution models (JSDM) to data collected by a scientific bottom trawl survey conducted between 1996 and 2019 (Fig. 1). We fitted separate models to analyze the presence-absence of the species and the abundance conditional on species presence (hereafter conditional abundance), and visualized their response to environmental factors (i.e., their niches) and trait-environment relationships by extracting the slope parameters. We determined the relative importance of environmental covariates and traits by partitioning the explained variation among fixed and random effects and assessed the strength of the phylogenetic signal. To gain further insight into community changes, we used the fitted models to predict expected species abundance on a spatial grid, calculated indices of abundance, and computed community-weighted mean traits.

## Results

Between 1996 and 2019, more than 4000 bottom trawl hauls were conducted in the North-Central Adriatic Sea, which led to the identification of 29 elasmobranch species. However, further analysis revealed that only 11 of these species were sufficiently represented in the data, and from those, only 9 were ultimately included in the analysis (for more details on the criteria for species selection, refer to the Methods section). These retained species have varying ages at first maturity, ranging from 2.5 to 15 years (mean: 7.1 years, s.d.: 4.4), and a mid-ranking trophic level (mean: 3.78, s.d.: 0.26). Four of the species exhibited viviparity, giving birth to live young, while the other five displayed oviparity, laying eggs as their reproductive mode (Table 1). Considering the entire survey period, the most dominant species were the small-spotted catshark being recorded in one-fourth of all hauls, followed by brown skate, spurdog, thornback skate and smooth-hound, which occurred in roughly 10% of the hauls. The remaining species occurred more sporadically (Table 1).

**Table 1:** Frequency of occurrence (percentage of trawl hauls where the species was caught), total number of individuals caught in all the trawl hauls, as well as traits values and classifications of the elasmobranchs included in the study.

Species	Frequency of occurrence (%)	Total number of individuals	Age at first maturity	Reproductive mode	Trophic level
Small-spotted catshark (*Scyliorhinus canicula*)	25.75	12,628	5.0^75^	Oviparous^75^	3.60^38^
Brown skate (*Raja miraletus*)	13.33	3124	2.5^73^	Oviparous^75^	3.67^76^
Spurdog (*Squalus acanthias*)	11.68	3068	15.0^73^	Viviparous^75^	3.90^38^
Thornback skate (*Raja clavata*)	10.74	1188	7.5^73^	Oviparous^75^	3.69^76^
Smooth-hound (*Mustelus* spp.)	7.75	1970	10.5^73^	Viviparous^75^	3.80^38^
Common eagle ray (*Myliobatis aquila*)	6.07	1065	3.0^73^	Viviparous^75^	3.33^39^
Marbled torpedo (*Torpedo marmorata*)	2.94	150	12.0^73^	Viviparous^75^	4.24^39^
Starry skate (*Raja asterias*)	2.62	169	3.5^73^	Oviparous^75^	3.82^75^
Nursehound (*Scyliorhinus stellaris*)	2.62	255	5.0^75^	Oviparous^75^	4.00^37^

Species are sorted in descending order according to their frequency of occurrence.

### Species modeling

The presence-absence (PA) and the conditional abundance (ABU) models showed a satisfactory fit to the data. The mean Tjur’s R$^2$ (AUC) was 0.29 (0.92) considering the PA model, whereas the explanatory power R$^2$ for the ABU model was on average 0.39 (Supplementary Fig. S2). Both species presence-absence and species conditional abundances were influenced by environmental variables. Variance partitioning over the explanatory variables included in the models showed that depth explained a substantial amount of variation (17% averaged over the species) among the fixed effects in the PA model, whereas in the ABU model, depth and bottom temperature explained on average 30% and 14% of all variation, respectively (Supplementary Fig. S3). Haul-level spatial random effects accounted for the largest portion of variance in both the PA and in the ABU models, explaining on average 69% and 32% of the variance, respectively. Temporal random effects, on the other hand, explained on average 9% of the total variation for the PA model and 12% for the ABU model (Supplementary Fig. S3).

**Figure 2:**
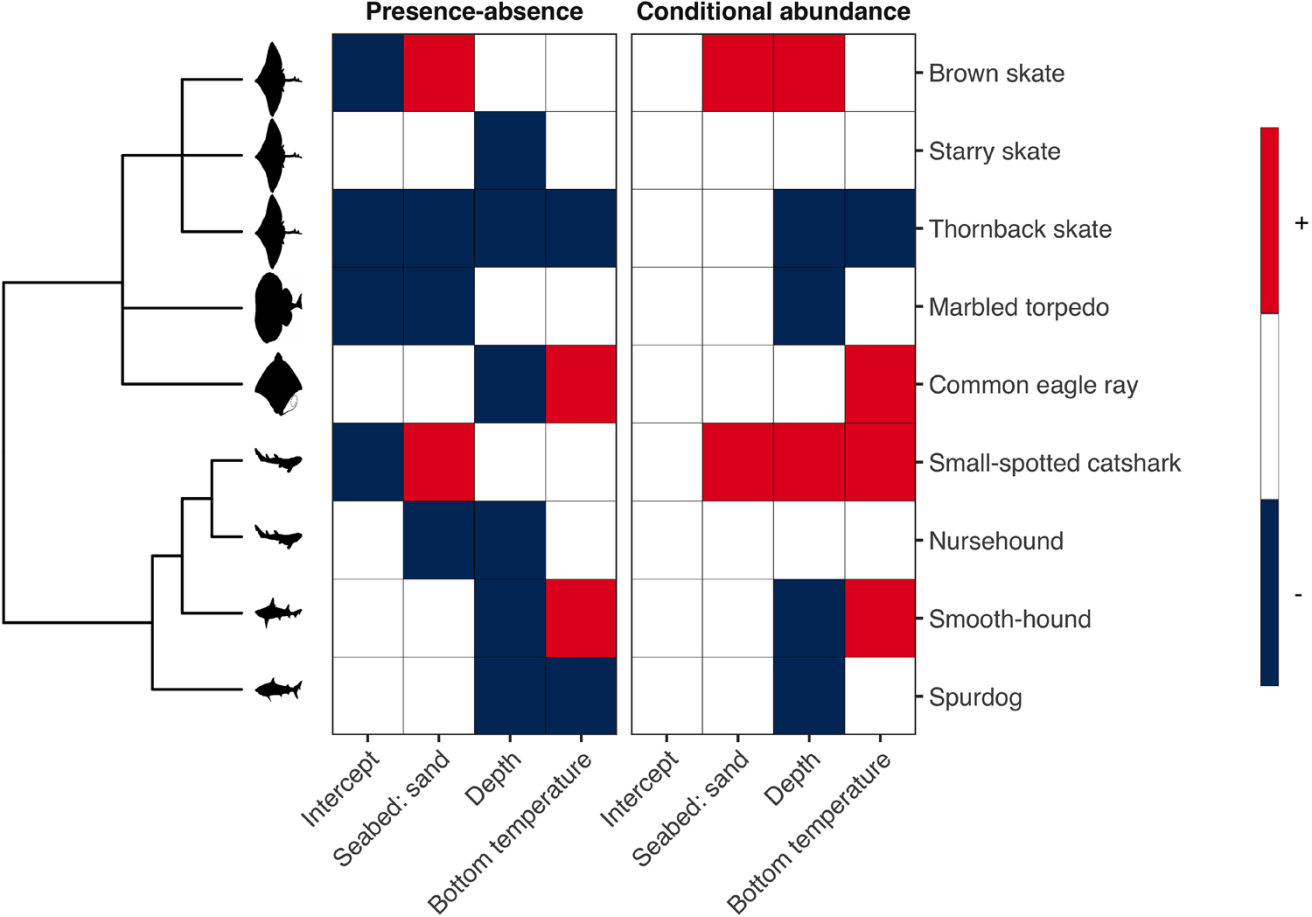
Heatmap of the estimated $\beta$ parameters linking the responses of species to environmental covariates. Responses that are positive with at least 95% posterior probability are shown in red, responses that are negative with at least 95% posterior probability are shown in blue, while responses that did not gain strong statistical support are shown in white. The intercept represents the average value of the response variable for the reference group of the seabed substrate covariate (i.e., seabed characterized by mud to muddy sand). For the seabed covariate, colors represent contrasts between mud to muddy sand and sand substrates such that red indicates species preferring the sand substrate over the mud to muddy sand, while blue the opposite. Species are ordered according to their phylogeny as illustrated by the phylogenetic tree shown on the left. Icon credits: PhyloPic (phylopic.org).

We found strong support for species-specific responses to the environmental covariates that in part differed between explaining the presence of a species (PA model) and the abundance of a species (ABU model) (Fig. 2). For instance, seabed type was important for determining if a species was present or not, while bottom temperature had a similar effect on both presence and abundance. Certain elasmobranchs species were more likely to occur in specific seabed types, such as brown skate and small-spotted catshark on sand seabeds and thornback skate, marbled torpedo, and nursehound on mud and muddy sand bottoms. Generally, most species had a lower occurrence probability with increasing depth. Warmer bottom temperature seemed to negatively affect certain species, while positively affecting others (Fig. 2). The species responses to environmental covariates showed a weak phylogenetic signal in the PA model (E[$\rho$] = 0.46; Pr[$\rho>$ 0] = 0.71) and a strong signal in the ABU model (E[$\rho$] = 0.86; Pr[$\rho>$ 0] = 0.97).

**Figure 3:**
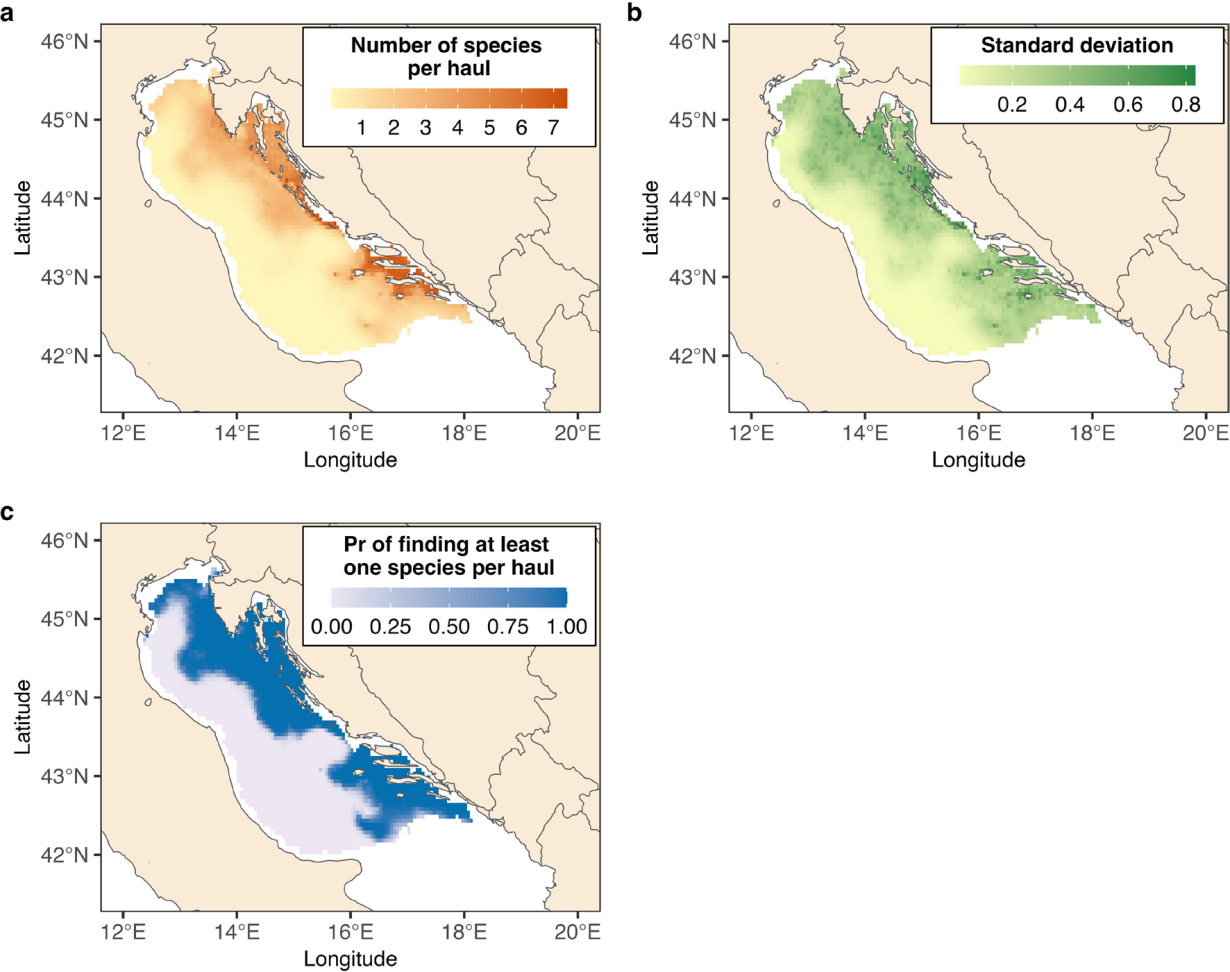
Predicted community features across the study area. Panel (**a**) shows the posterior mean number of species per haul, panel (**b**) the posterior standard deviation of the mean number of species per haul, and panel (**c**) the posterior probability (Pr) of finding at least one species per haul. Predictions refer to the year 2019. In the predictions, the swept area is set to a constant value equal to the mean of the trawl hauls used in the study (i.e., 0.047 km$^2$).

The models’ predictions identified a clear spatial pattern in the elasmobranch community of the North-Central Adriatic Sea (Fig. 3). Specifically, species diversity was significantly lower in the western part of the study area, while the central and southern Croatian coasts in the eastern part exhibited higher diversity (Fig. 3a). The level of uncertainty in the models’ predictions was higher in the eastern part of the study area but overall relatively low, with a margin of error of less than one species (Fig. 3b). Likewise, species had higher probability of occurrence and were more abundant along the Croatian coasts (Fig. 3c, Supplementary Fig. S4). Notably, the northernmost part of the basin showed a relatively high abundance of common eagle ray and smooth-hound, despite lower overall species diversity in this area (Supplementary Fig. S4, Fig. 3a)

**Figure 4:**
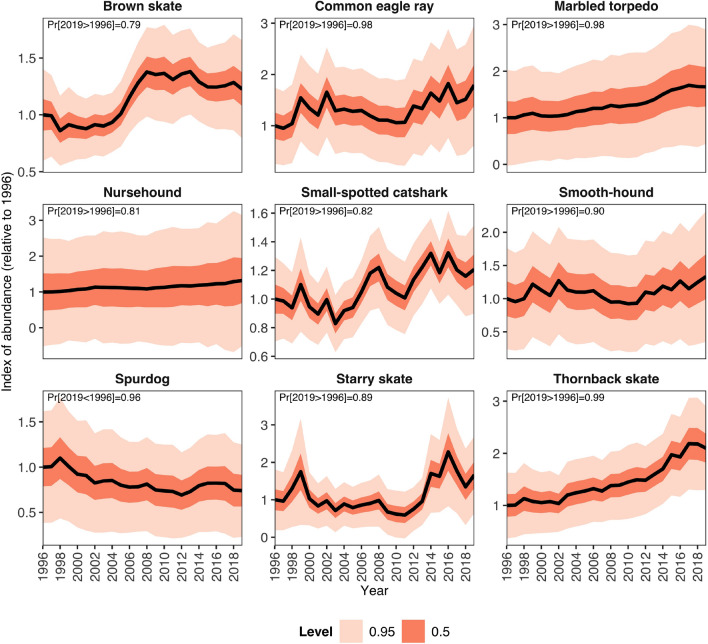
Indices of abundance. Black lines represent the posterior mean estimate while the dark and light red areas represent 50% and 95% credible interval levels, respectively. For each species, the abundance index is scaled to 1996 posterior mean values (i.e., a value of 2 on the *y*-axis indicates 2 times higher abundance than in 1996). In the top-left part of each panel, in case of increasing trend, the probability (Pr) by which the last year (2019) has higher estimated abundance compared to the first year of the survey (1996) is reported; in case of decreasing trend, the probability by which the last year has lower estimated abundance compared to the first year.

Over the study period, we predicted the relative temporal changes in species abundances (Fig. 4). The abundance index showed a general upward trend for the majority of the species. In particular, the thornback skate recorded the highest abundance increase (110%) comparing 1996 to 2019, followed by common eagle ray (80%), marbled torpedo (66%), and starry skate (66%). Whereas spurdog was the only elasmobranch decreasing (− 26%).

### Traits modeling

**Figure 5:**
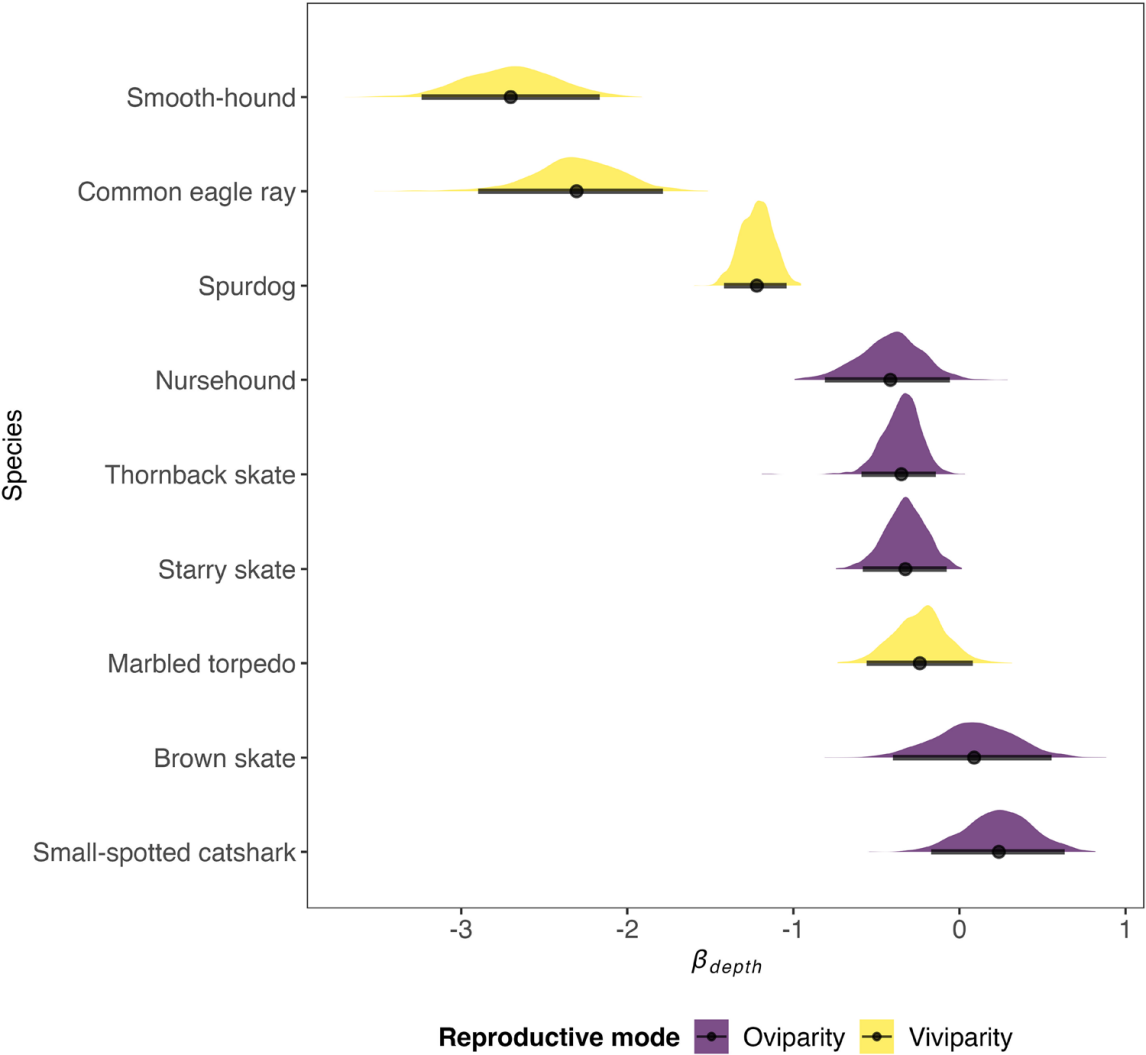
Posterior distribution of the $\beta _{depth}$ parameters linking species occurrences to log-transformed depth. The point intervals represent the mean values and the 95% credible intervals. Above the point intervals the probability density functions are plotted (oviparous and viviparous species shown in violet and yellow, respectively). Lower values indicate stronger species preferences for shallower waters. Species are ordered according to mean values in ascending order.

The selected ecological traits (i.e., age at fist maturity, reproductive mode, and trophic level) explained 44% of the variation in species occurrence and 40% of the variation in species abundance. Considering species niches (i.e., species responses to environmental covariates), traits explained 52% (PA model) and 66% (ABU model) of the species response to depth, 28% (PA model) and 20% (ABU model) of the species response to the seabed substrate, 19% (PA model) and 30% (ABU model) of the species response to bottom temperature. Considering the PA model, the $\gamma$ parameters (i.e., the parameters linking species traits to species niches) indicated that viviparity is negatively correlated with depth (with at least 95% posterior support; Supplementary Fig. S5). This relationship became more obvious once clustering the species responses to depth ($\beta _{depth}$) by reproductive mode which shows that viviparous are more negatively affected by depth (i.e., lower $\beta _{depth}$ values) (Fig. 5). Contrastingly, we found no significant relationship between trait and environmental covariates for the ABU model (Supplementary Fig. S5).

**Figure 6:**
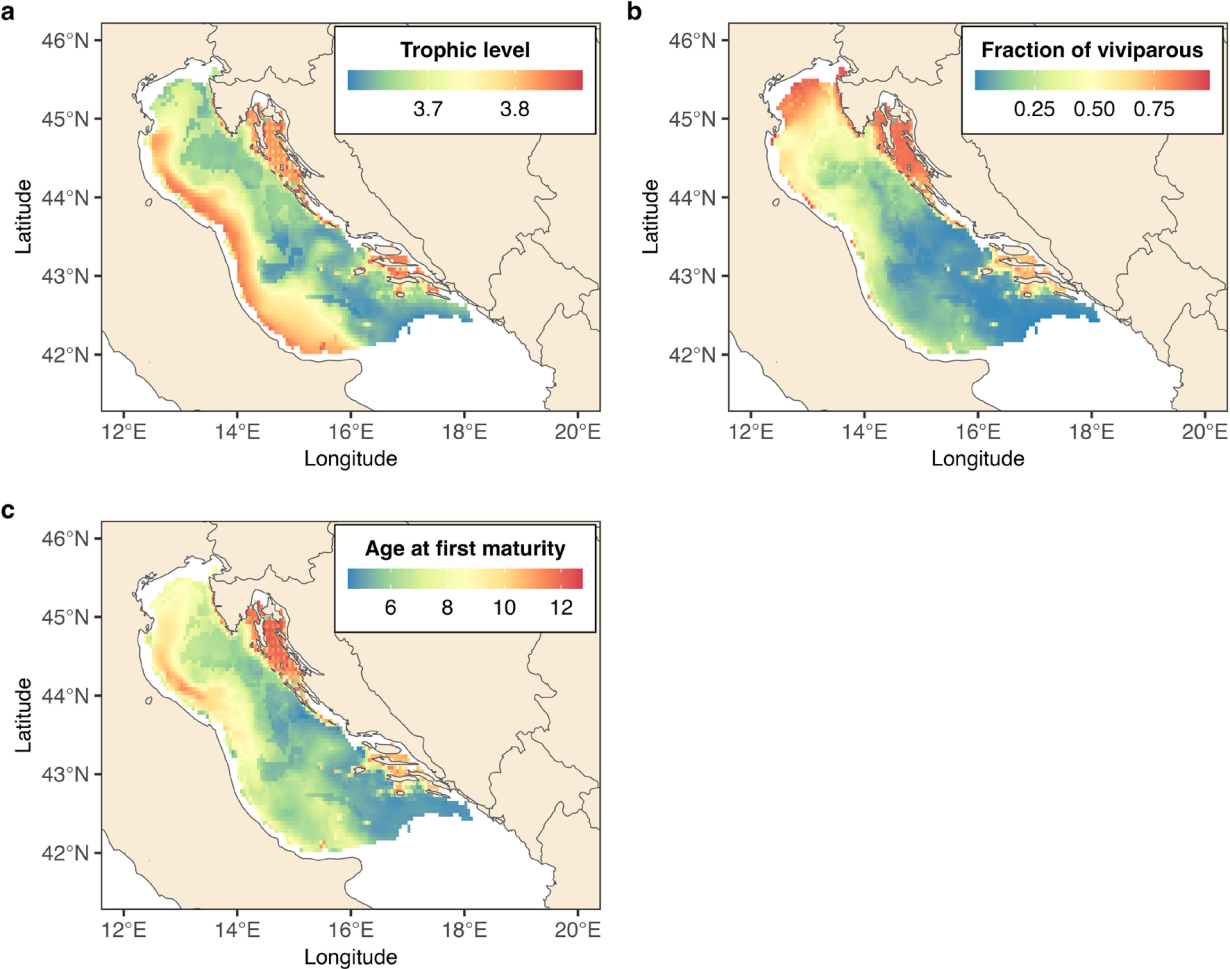
Predicted community-weighted mean traits. Panel (**a**) shows the posterior mean estimate of the trophic level, panel (**b**) the posterior mean estimate of the fraction of viviparous species, and panel (**c**) the posterior mean estimate of the age at first maturity. Predictions refer to the year 2019.

To generate community-weighted mean (CWM) trait maps we weighted trait values by species relative abundances. These maps indicate higher CWM trophic level along the Italian and Croatian central and southern coastal areas (Fig. 6). Also, the shallow areas along the northern and eastern coasts encompassed a higher fraction of viviparous species compared to the deeper southern areas that were dominated by oviparous species such as thornback skate, brown skate, and small-spotted catshark (Supplementary Fig. S4). Finally, the predicted CWM age at first maturity was higher along the Croatian coast, whereas lower values were present in the deeper southern areas.

**Figure 7:**
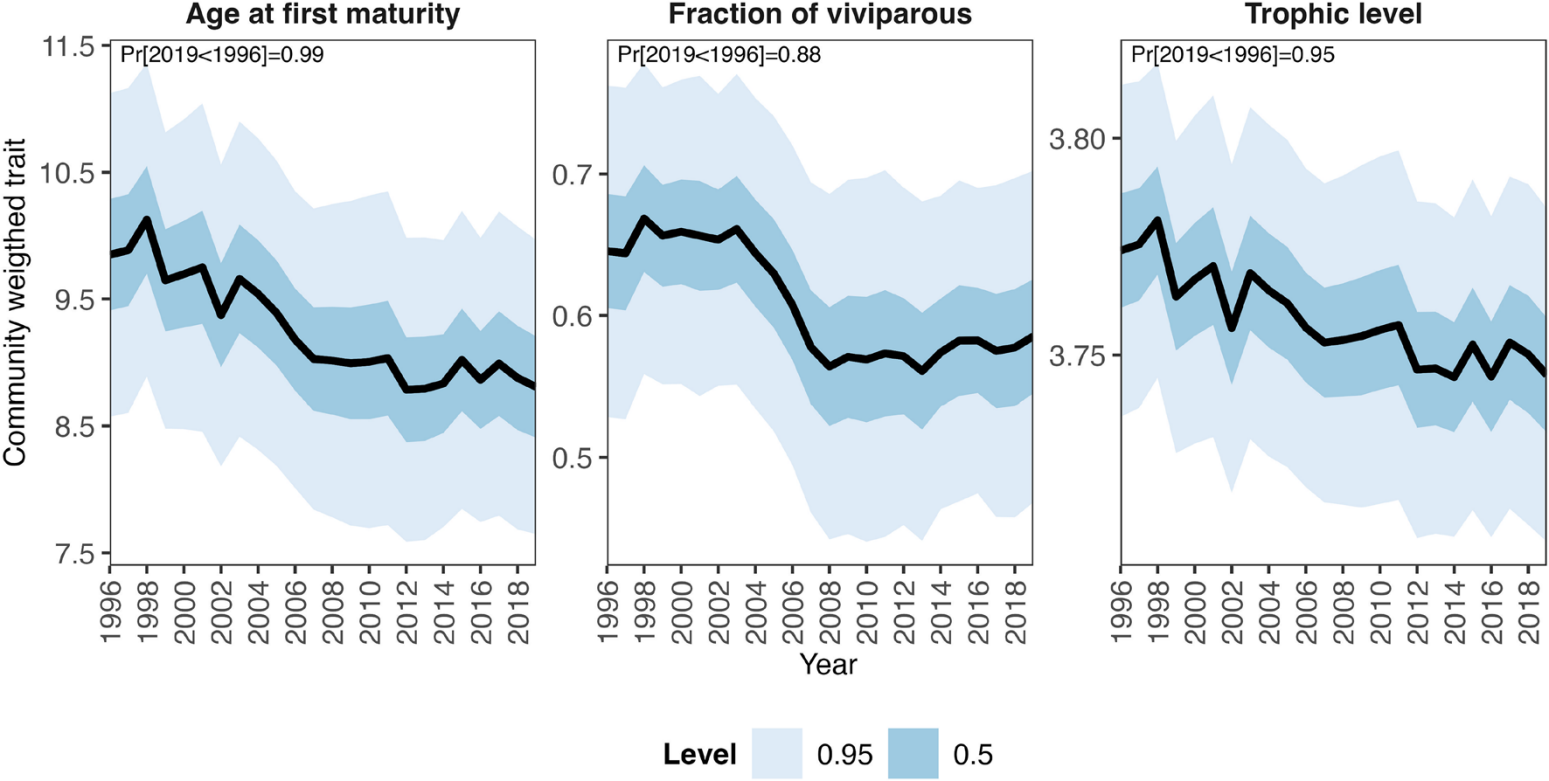
Predicted community-weighted trend of trait categories. Black lines represent the posterior mean estimate, while the dark and light blue areas represent 50% and 95% credible interval levels, respectively. In the top-left part of each panel is reported the probability (Pr) by which the last year (2019) has a lower estimated community-weighted mean trait value compared to the first year (1996). Note the different scale of the *y*-axes.

Our modeling results estimated an overall decline in community-weighted mean traits values considering age at first maturity, fraction of viviparous species and trophic level over time. Examining the magnitude of the decline, CWM age at first maturity declined by 11%, the fraction of viviparous species by 9%, while CWM trophic level declined by less than 1%, from 1996 to 2019 (Fig. 7).

## Discussion

A growing body of literature reports the decline of elasmobranchs in coastal and oceanic ecosystems worldwide^5,8,42^. However, understanding changes in community composition is challenging due to multiple factors that influence populations, such as environmental conditions, biotic interactions, and stochastic events such as colonization, extinction, ecological drift, and environmental stochasticity. Insights into community dynamics can be gained from the incorporation of traits, phylogeny, and by explicitly acknowledging co-occurence patterns among species. Our study takes a leap forward towards a mechanistic understanding of the spatio-temporal changes of an elasmobranch community over two decades in the North-Central Adriatic Sea. Here, by fitting joint species distribution models we show that the Adriatic elasmobranch community is primarily sorted by space and depth, which is in agreement with studies performed in other areas^43^. We show that the eastern part of the basin has higher abundance and diversity compared to the western part. Secondly, we present species-specific abundance trends which show an increase for all species analysed but spurdog, which declined in abundance. Thirdly, we highlight that the included ecological traits (i.e., trophic level, age at first maturity and reproductive mode) explain about half of the variation in species occurrence and abundance as well as of the response to environmental covariates, suggesting that the use of traits is beneficial in modelling these rare species.

Our results show a higher abundance and diversity of elasmobranchs in the eastern Adriatic Sea, supporting previous findings^7,35^. This spatial pattern has been linked in literature to past and recent differences in the fishing effort between the Italian and Croatian fleets^7^ (see Supplementary Fig. S6 for a spatial effort projection), although depth, temperature, seafloor morphology and sea bed type have been suggested as well to be important factors influencing elasmobranch distribution in the Mediterranean Sea (e.g., this study,^7,44^). Previous studies^7,35^ covering large parts of the Adriatic Sea depicted a decline of more than 90% in elasmobranch abundance comparing the late 1940s with the early 2000s levels. The most dramatic decline was recorded for the thornback skate and the small-spotted catshark^7^. Yet, few species showed departures from this general trend such as brown skate, common eagle ray, marbled torpedo, and spurdog, which instead increased^7^. With our data covering the period 1996-2019, we extend by 14 years the previous analysis^7^ which presented elasmobranch trends estimated from the MEDITS trawl survey for the period 1994-2005. We found a strong rebound of the thornback skate, which doubled in abundance comparing the 1996 with the 2019. This is consistent with recent trends observed by fishers in the Adriatic basin, who have reported an increase in the occurrence of *Raja* spp. over the last decade^27^. These observations are further corroborated by an increase in the abundance of this species complex in commercial landings at Chioggia harbor^45^, which is among the primary fishing ports in the Adriatic Sea. In continuation with what was previously detected^7^, we estimated an increase in abundance for the common eagle ray (1.8 times) and the marbled torpedo (1.7 times). We found also an increase, although less pronounced, in the abundance of the starry skate, the smooth-hound, the nursehound, the brown skate, and the small-spotted catshark. These species showed either a continuous positive trend from the mid 1990s or an increase in the most recent years. Similarly to our results, recent increase in abundance for thornback skate and small-spotted catshark were also detected in the South of Sicily^46,47^, in the Western Mediterranean^48^, and in the North Sea^49^, suggesting convergent patterns in historically heavily exploited areas that have experienced recent reduction in fishing effort. A negative trend since 1994 was previously detected for spurdog^7^ and our results show that its abundance remained steady at a low level in the past two decades. Yet, this species is listed as Endangered in the Mediterranean by the International Union for Conservation of Nature (IUCN)^50^ and alarmingly our study reveals that it has not shown signs of recovery in the Adriatic Sea. A decline in abundance for spurdog was also evident in the North Sea^49^ from the late 1970s, which may indicate that the current level of fishing effort in the European waters did not allow for the rebound of this species.

Despite including only 3 ecological traits in the presence-absence (PA) model and in the abundance conditional on presence (ABU) model, they explained 44% and 40% of the variation in the species responses to the covariates considered in the analysis, respectively. Although this might be suggestive of the relevance of the traits included to the community assembly processes we also note that life-histories (such as reproductive mode and age at first maturity included in the analysis) are constrained by trade-offs which mainly co-vary along a ‘fast-slow continuum’^51^. At the fast extreme, species develop quicker, are more productive but have shorter life spans; whereas at the slow extreme, species have higher survival rates, develop slower and live longer. It follows that, for instance, age at first maturity is linked with other relevant life-history traits such as total length, average adult life expectancy and species growth rate^3^. However, among the trait-environment relationships we examined, the reproductive mode was tightly linked with depth which may indicate that environmental filtering was at play. Our study indicates that viviparous elasmobranch species are typically found in shallower waters compared to oviparous species, likely due to several factors. Firstly, the reproductive strategy of viviparous species involves giving birth to live offspring that are larger in size, which may be less effective in deeper waters where there is limited prey availability to support their growth and development. Additionally, shallower waters provide more favorable conditions for the development and survival of the young, including warmer temperatures and hiding places to protect them from predators^52^. Furthermore, viviparous elasmobranchs are on average larger bodied than oviparous relatives^53^, which enable greater active dispersal ability and wider home ranges^54^ , enhancing their ability to persist in heavily exploited shallow areas. Based on our findings, we observed that the elasmobranch community in the Adriatic Sea is segregated along a depth gradient, with viviparous species such as spurdog, smooth-hound, and common eagle ray primarily occupying coastal shallow areas, while oviparous species reside also in the deeper areas. Additionally, we identified a strong spatial pattern in the predicted community-weighted mean traits, showing a decreasing gradient of viviparous species from the northern and eastern coastal to offshore areas. Furthermore, we found that higher trophic levels and late-maturing species tend to concentrate along the eastern costal areas, but also in the western coastal areas despite the low diversity and abundance of species in this region. Among the included traits, we found a noticeable decline over time of the community-weighted age at first maturity, the fraction of viviparous species and the trophic level, although the decline of the latter was minor. The degree of vulnerability of shark and rays species is known to be associated with life history traits, such that for instance species exhibiting late maturation and viviparity are often the most vulnerable to exploitation^1,3,4,55^. The strong increase from the late 1990s of oviparous or early-maturing species such as the thornback skate and the common eagle ray, as well as the decline of the late-maturing viviparous spurdog, found in our study are in accordance with this expectation. However, it is important to note that the CWM traits are primarily influenced by the most abundant species, such as the small-spotted catshark, brown skate, thornback skate, and spurdog, rather than rare ones like the marbled torpedo, which is also a late-maturing viviparous species that has experienced a substantial increase. This pattern suggests that the EU’s fleet decommissioning scheme, aimed at promoting sustainable European fisheries^31,32^, may have benefited more resilient species, while less resilient ones may have struggled to recover. Nonetheless, other factors such as historical removal of large predators of these elasmobranchs^56^ or the availability of open ecological niches resulting from the disappearance of other elasmobranch species^7^ could also have played a role in driving the observed changes in the community.

The high value of the estimated phylogenetic signals in the ABU model suggests the existence of some traits correlated with phylogeny that were not considered in our analysis, and which may therefore contribute to explain the abundance of species in a certain environment. Therefore, we suggest that further research is needed to identify other traits than those considered here.

In conclusion, our study including ecological traits provided a helpful framework for predicting the structure and dynamics of an elasmobranch community in the Adriatic Sea over the past three decades. Our findings indicate a lower age at first maturity and a decreased fraction of viviparous species in the current community compared to the community observed in the late 1990s. Furthermore, we observed a concerning and persistent decline in the threatened spurdog population, while most other elasmobranch species in the basin experienced an increase. The elasmobranch community in the Adriatic Sea has been subjected to multiple population depletions over time^26^, starting with the decline of large predatory sharks in the 19th and 20th centuries^25,56^. During the 20th century, numerous pelagic and demersal sharks, which were once widespread, experienced declines or even disappeared altogether in this basin^7,57^. Therefore, caution is necessary when interpreting recent increases in the abundance of some species as an early indication of recovery, since their recent rebounds are still far from their historical baselines.

## Methods

### Study area

The North-Central Adriatic Sea is a semi-enclosed basin corresponding to the FAO - Geographical Sub-Area 17 (GSA 17) located in the Central Mediterranean, between the Italian peninsula and the Balkans (Fig. 1). This basin is a shallow and mainly eutrophic sea with clear morphological differences along both the longitudinal and the latitudinal axes. Most of the basin is characterized by a wide continental shelf where depth gradually increases from north to south. However, the central area is characterized by meso-Adriatic depressions (Pomo/Jabuka Pit), where the depth reaches $\sim$ 270 m. The sea bottom is mostly covered with recent muddy and coastal sandy sediments supplied by rivers and dispersed southward by currents while no present sedimentation affects the offshore seabed in the Northern Adriatic, which is characterized by relict sand^58^. The eastern coast is rocky and presents numerous islands, whereas the western coast is flat, alluvial, and characterized by heavy river runoff. The Po and other northern Italian rivers contribute to approximately 20% of the whole Mediterranean river runoff^59^ and introduce large fluxes of nutrients^60^, making this basin the most productive area of the Mediterranean Sea^61^, and consequently one of the most intensively fished areas in Europe^62^. The general pattern of water circulation is cyclonic with water masses inflow from the Eastern Mediterranean along the eastern side of the Adriatic Sea and outflow along the western side. The Adriatic Sea circulation and water masses are also strongly influenced by river runoff and atmospheric conditions which in turn affect salinity and temperature^63^. Pronounced seasonal fluctuations in environmental conditions, cause high seasonal variations in coastal waters, where bottom temperatures range from 7$^{\circ }$ in winter to 27$^{\circ }$C in summer^64^. Contrarily, the thermal variability of the deeper areas is less pronounced, with values ranging between 10$^{\circ }$C in winter and 18$^{\circ }$C in summer at a depth of 50 m^64^.

### Survey data

The whole dataset comprised 4231 bottom trawl hauls performed during the Mediterranean International Trawl Survey (MEDITS)^65^, which is jointly performed by the Laboratory of Marine Biology and Fisheries of Fano (Italy) and the Institute of Oceanography and Fisheries of Split (Croatia) on an annual basis since 1996 generally in the late spring-summer period (May to September). Hauls were located from 10 to 500 m of depth, following a random-stratified sampling scheme where strata were defined according to depth. The sampling gear utilized was the GOC-73 experimental bottom trawl, which featured a horizontal opening of 16-22 meters and a vertical opening of approximately 2.4 meters. The codend of the trawl was equipped with a 20mm side diamond stretched mesh. It should be noted, however, that potential biases in capturing elasmobranchs may exist for bottom trawls. For instance, skates, have a body morphology that keeps them close to the sea bed making them more likely to avoid the trawl entrance^66^. Additionally, some species of medium to large-sized sharks such as spurdog and smooth-hound are known for their fast swimming speeds and pelagic behavior^67^. These characteristics make them less likely to be captured by bottom trawl survey gear, which may result in an underestimation of their absolute abundance. Furthermore, the interpretation of trends in spurdog abundance may be complicated by their tendency to aggregate. However, the survey area have been overall covered consistently over the years, and our aim was to analyse the relative changes in species abundances and community composition, therefore we consider the results robust to these sampling caveats that are common in any trawl survey. Further details on sampling procedures, data collection and analysis can be found in the MEDITS handbook^68^. For this study, we excluded those hauls which have occurred in the autumn-winter period (October to December) due to the potential redistribution of elasmobranch among seasons^69^, leading to a total of 4050 trawl hauls analysed. The abundance (i.e., the number of fish per trawl haul) of 29 elasmobranch species was recorded totally. However, we excluded those species scarcely represented (i.e., that occurred in less than 2% of the trawl hauls or with less than 150 individuals in the whole dataset). The common smooth-hound *Mustelus mustelus*, and the blackspotted smooth-hound *Mustelus punctulatus* were grouped together under the category smooth-hound. The morphological identification for the *Mustelus* genus is not trivial^70^, and the common smooth-hound and the blackspotted smooth-hound can be often misidentified. Also, the two species co-occur in the North-Central Adriatic Sea, have a similar diet^71^, share similar habitats, and the chance of hybridization is not excluded^70,72^. The blackmouth shark *Galeus melastomus*, was excluded from the analysis because it inhabits deeper water^73^ and its distribution range is likely not adequately covered by the survey. As a result, our final dataset comprised the recorded abundance of nine elasmobranch species (Table 1).

### Environmental covariates

As explanatory covariates, we included log-transformed depth, and bottom temperature as linear fixed effects and seabed substrate as a binary categorical variable (sand, mud to muddy sand). Additionally, we accounted for the variability in the sampling effort including the log-transformed swept area of each haul as a predictor. To account for any underlying linear temporal trends that may have been present but not captured by the included covariates, we also added survey year as a linear fixed effect. We ensured that covariates were not strongly correlated with each other (r $< 0.7$) (Supplementary Fig. S7). We chose environmental variables based on the availability of the data and assumed relevance to the community assemblages. We extracted the monthly mean bottom temperature from the Copernicus Marine Service Data^74^ and derived seabed substrate from the European Marine Observation Data Network (EMODnet) Geology Project (http://www.emodnet-geology.eu/), using the Folk 5 classification. To integrate these environmental variables with the trawl hauls data, we associated each trawl haul with the nearest spatial point of the variables. In the case of bottom temperature, we also matched the data in time. For the grid of the spatio-temporal predictions, we followed the same approach but we used the mean values of bottom temperature over the survey period (May to September) in each cell. This step enabled us to standardize the effects of bottom temperature across different years.

### Traits and phylogeny

As species traits, we compiled data on three ecological traits related to life-histories and feeding habits that we expected to influence the spatio-temporal dynamics of the elasmobranchs, that were comparable across sharks and rays, and for which information was available in the literature. Included traits were age at first maturity, reproductive mode and trophic level (Table 1). Age at first maturity data were retrieved from a field guide to the Mediterranean elasmobranchs^73^ and complemented with a publicly available dataset on marine fish traits^75^. If data were disaggregated by sex we kept the female age at first maturity, and in case data were available as a range we took the median value. Wherever age at first maturity information was not available at the species level we used inferred data from the genus level (i.e., for small-spottedcatshark and nursehound). Considering trophic level, we sourced data from standardised diet studies^38,39,76^. For smooth-hound, we used trait data of *Mustelus mustelus*. We provide detailed references in Table 1. We further ensured that traits were not highly correlated (r $< 0.7$) (Supplementary Fig. S8). To account for phylogenetic dependencies, we included a phylogenetic correlation matrix in the model’s covariance structure. However, since true phylogenetic information of species was not available, we used the as.phylo function in the ape R-package^77^ to build a phylogenetic tree of the included species based on their taxonomic structure including phylum, class, order, family, genus and species, and assuming equal branch length of 1 between each node. This allowed the model to estimate how much of the residual environmental responses of the species were explained by phylogenetic correlations. The strength of the phylogenetic signal is measured by the parameter $\rho$ which is constrained between 0 and 1. A value of $\rho$ = 0 indicates that the residual variance in species’ environmental niches is independent among the species, while when $\rho$ = 1, species’ environmental niches are fully explained by their phylogeny.

### Statistical analysis

Using the ‘Hmsc’ R-package^22,41^ version 3.0-12 we fitted joint species distribution models (JSDMs) to the elasmobranch community abundance data while also including information on traits, environmental covariates and phylogenetic constraints. To account for the spatial and temporal stochasticity of the data, as well as to model residual co-occurrence among species, we employed spatially^78^ and temporally structured latent variables. These variables allowed us to incorporate hierarchical structures in our model and account for any unexplained variation in the data. We implemented spatially structured latent variables through the predictive Gaussian process for big spatial data^79^. The response matrix was the number of individuals of each elasmobranch species per trawl haul. Due to the zero-inflated nature of the data, we applied a hurdle approach, i.e., one model for presence-absence (PA) and another model for conditional abundance (ABU). We employed probit regression in the PA model and a log-linear regression for the abundance data in the ABU model. The abundance data in the ABU model were scaled to zero mean and unit variance within each species to better accommodate default priors, which are reported in the ‘Hmsc’ R-package paper^41^. For additional information on the model equations, please refer to the Supplementary Information. For each model, we sampled the posterior distribution with four Markov chains Monte Carlo (MCMC) simulations, each of which was run for 37,500 iterations, the first 12,500 being discarded as burn-in. The chains were thinned by 100, resulting in 250 posterior samples per chain, returning 1000 posterior samples in total. After fitting the model, we examined and ensured the convergence of the MCMC simulations by examining the potential scale reduction factors^80^ (Supplementary Figs. S9–S10). The explanatory power of the model was evaluated by computing the coefficient of discrimination Tjur’s R$^2$^81^ and the Area Under the Curve (AUC)^82^ for the PA model, which measures how well the model discriminates between presences and absence, and by computing the R$^2$ for the ABU model. The overall explanatory power of each model was then summarized as the mean explanatory power across species. To visualize the species niches, we extracted from the models fit the so-called $\beta$ parameters (regression slopes) which represent species-specific responses to environmental covariates. To visualize the trait-environment relationships, we extracted from the models fit the so-called $\gamma$ parameters which measure the influences of the traits on the species’ responses to the environmental covariates. To determine the relative importance of the environmental covariates and to measure how much of the variation in species occurrences and abundances are explained by their traits we partitioned the explained variation among the fixed and random effects^22,83^. We then used the parameterized model to predict the expected species abundance on a 0.05$^{\circ }$ latitude x 0.05$^{\circ }$ longitude grid. The abundance of each species was computed as the product of occurrence probability (from the PA model) and abundance conditional on presence (from the ABU model). In these predictions, we fixed the swept area value to the mean of the trawl hauls used in this study (i.e., 0.047 km$^2$). To functionally characterize the elasmobranch community, we then computed community-weighted mean traits (i.e., haul-level trait values weighted by species abundances) on predicted abundances. Indices of abundance were computed by raising the predicted abundance to the cell area and summing across the grid cells. The mean and the credible intervals were reported as summary statistics. All analysis was performed in R^84^ version 4.0.4.

## Supplementary Information

Supplementary Information.

## Data Availability

The survey data that support the findings of this study were collected within the EU Data collection Framework (DCF - MEDITS) and are available upon official request to the Direzione Generale della Pesca Marittima e dell’Acquacoltura of the Ministero dell’Agricoltura, della Sovranità Alimentare e delle Foreste (MASAF) and to the Croatian Ministry of Agriculture.
