# Supplementary material for: Influence of ecological traits on spatio-temporal dynamics of an elasmobranch community in a heavily exploited basin
Source: Sci Rep. 2023 Jun 13;13:9596. doi: 10.1038/s41598-023-36038-y (PMC10264359; doi:10.1038/s41598-023-36038-y)
Supplement: Supplementary file 1 — Supplementary Information. [file 41598_2023_36038_MOESM1_ESM.pdf]

Supplementary Information

Influence of ecological traits on spatio-temporal dynamics of an elasmobranch community in a heavily exploited basin

Federico Maioli[1,*], Benjamin Weigel[2,3], Elettra Chiarabelli[1,4,5], Chiara Manfredi[1], Alessandra Anibaldi[1,4], Igor Isailović[6], Nedo Vrgoč[6], and Michele Casini[1,7,*]

[1]University of Bologna, Department of Biological, Geological, and Environmental Sciences, Laboratory of Marine Biology and Fisheries, Fano, 61032, Italy
[2]University of Helsinki, Faculty of Biological and Environmental Sciences, Organismal and Evolutionary Biology Research Programme, Research Centre for Ecological Change, Helsinki, 00100, Finland
[3]INRAE, EABX, Cestas, 33612, France
[4]CoNISMa, Rome, 00196, Italy
[5]University of Trieste, Department of Life Sciences, Trieste, 34127, Italy
[6]Institute of Oceanography and Fisheries, Split, 21000, Croatia
[7]Swedish University of Agricultural Sciences, Department of Aquatic Resources, Institute of Marine Research, Lysekil, 45330, Sweden
[*]Corresponding authors: f.maioli@unibo.it; m.casini@unibo.it; michele.casini@slu.se

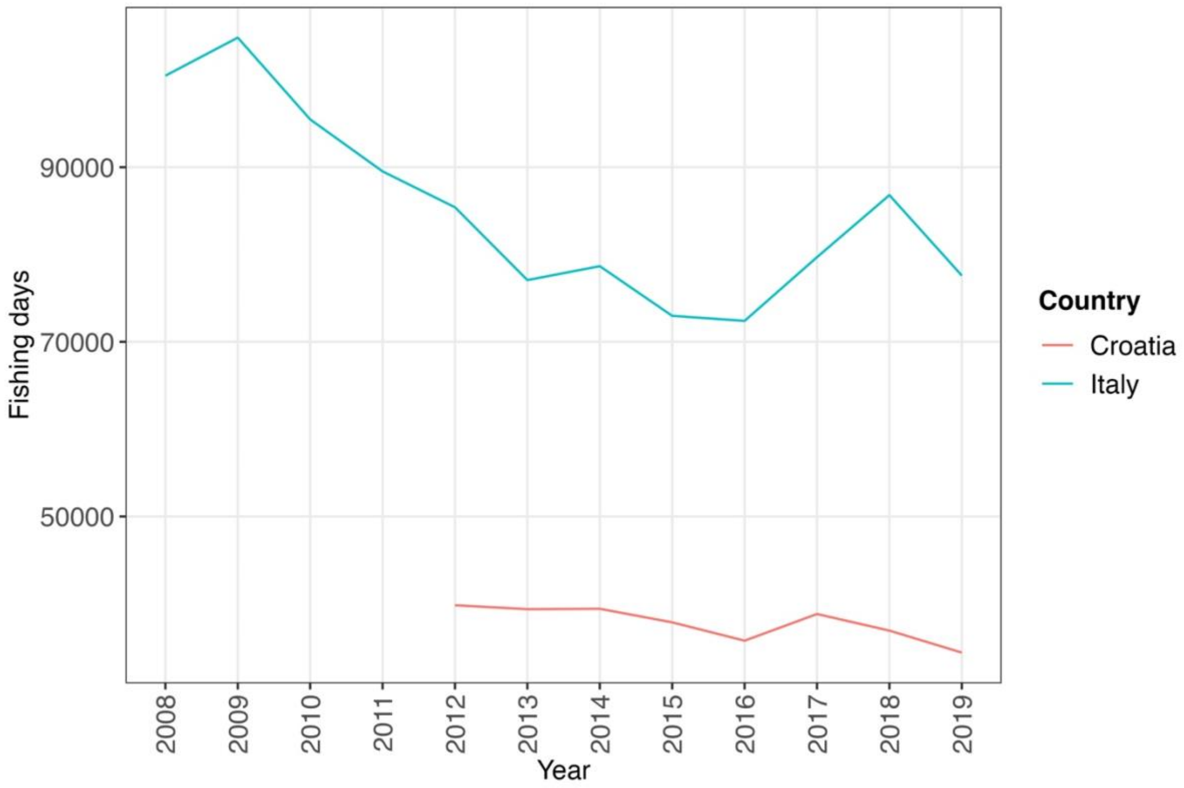

Figure S1. Trend in fishing effort in the North-Central Adriatic Sea from 2008 to 2019. Data are obtained from the Scientific, Technical and Economic Committee for Fisheries (STECF; https://stecf.jrc.ec.europa.eu/reports/economic). The data are filtered for beam trawlers (TBB), and demersal trawlers (DTS). Fishing effort is expressed as the number of days fishing vessels spent fishing.

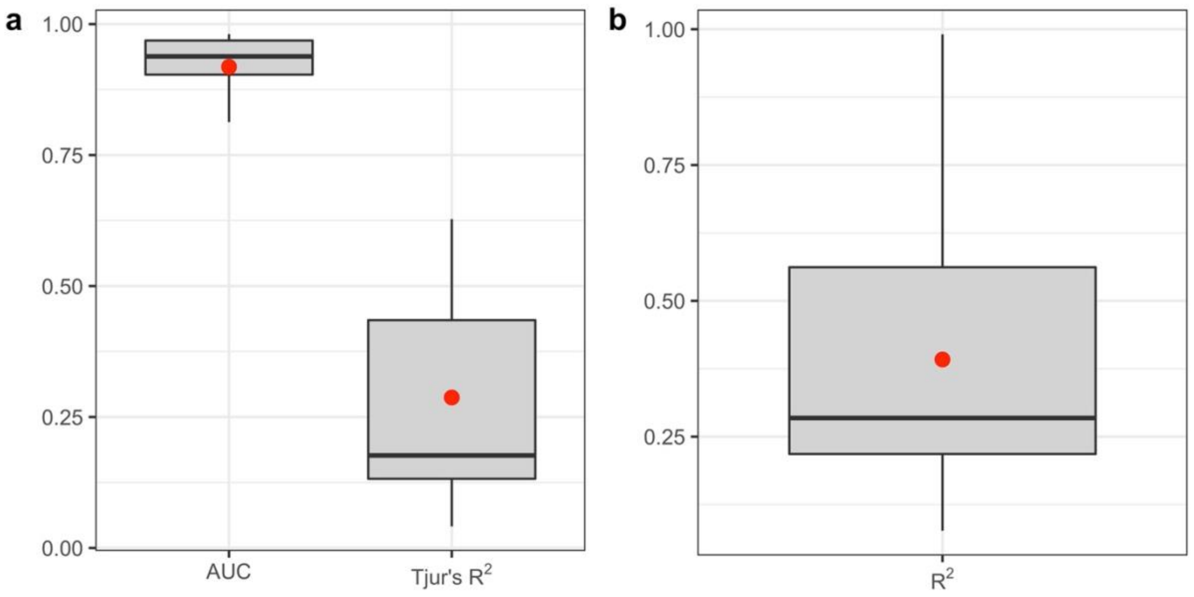

Figure S2. Boxplots showing the explanatory power. The panels show the results (a) for the presence-absence (PA) model and (b) for the conditional abundance (ABU) model. Data point represents species-specific values. Red dots represent mean values.

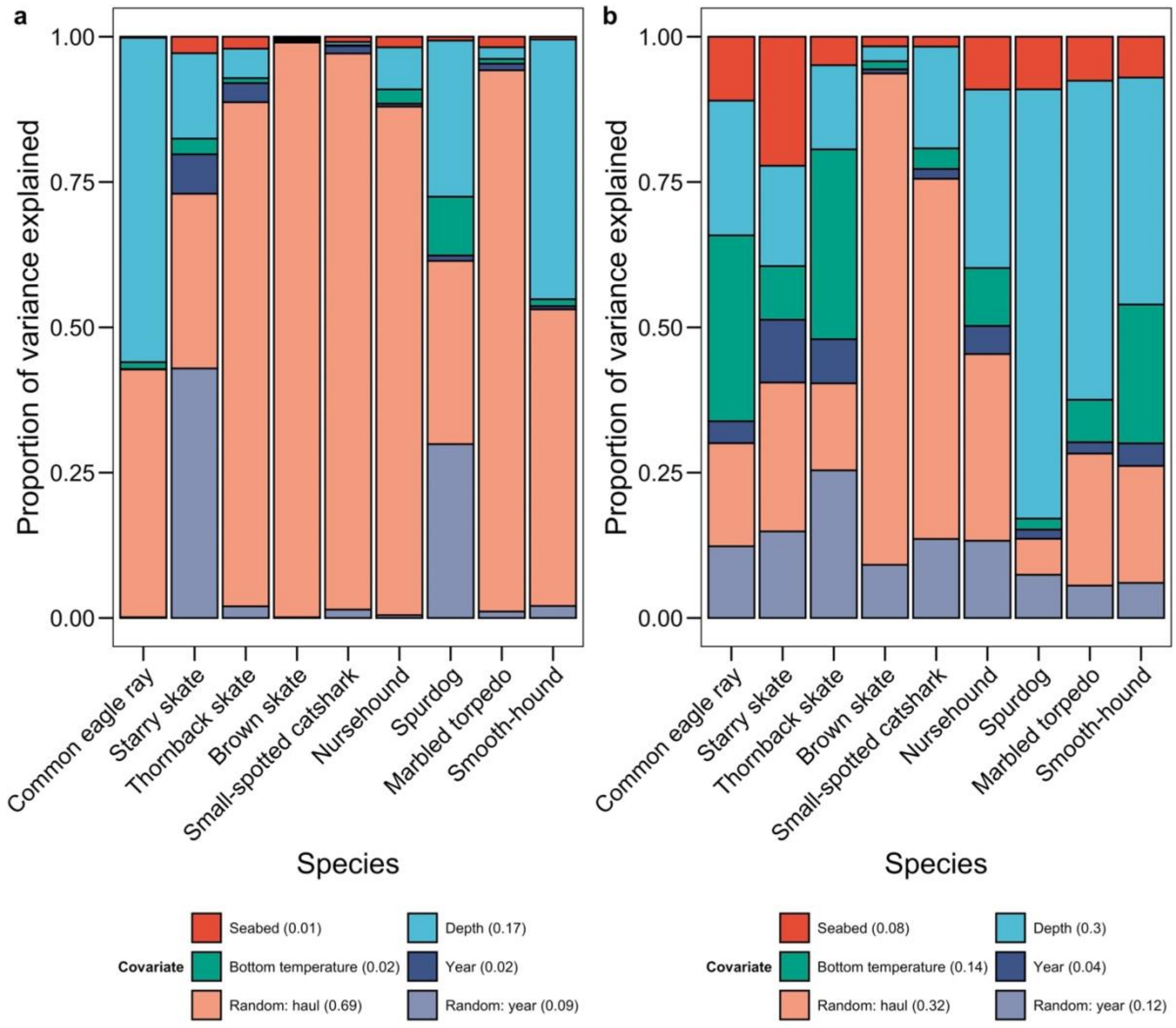

Figure S3. Variance partitioning among the explanatory variables included in the models. The panels show the results (a) for the presence-absence model and (b) for the conditional abundance model. The explanatory power is measured by Tjur R^2 for the presence-absence (PA) model and R^2 for the conditional abundance (ABU) model. The mean variance proportions averaged over the species are reported in brackets alongside the legend. 'Random: haul' indicates haul-level spatial random effects, while 'Random: year' represents temporal random effects.

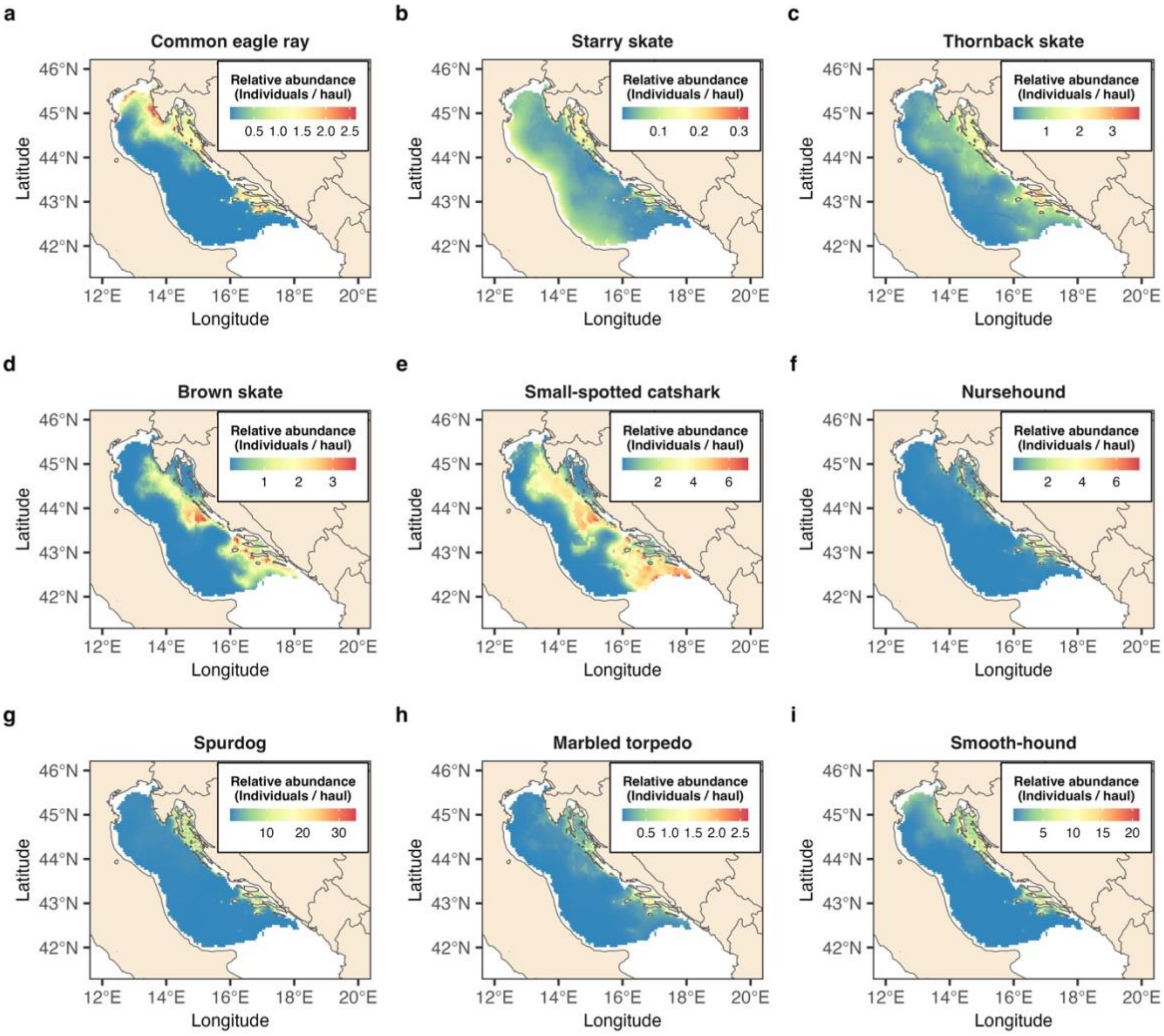

Figure S4. Predicted species mean abundance across the study area. Predictions refer to the year 2019. In the predictions, the swept area value is fixed to the mean of the trawl hauls used in the study (i.e., 0.047 km^2).

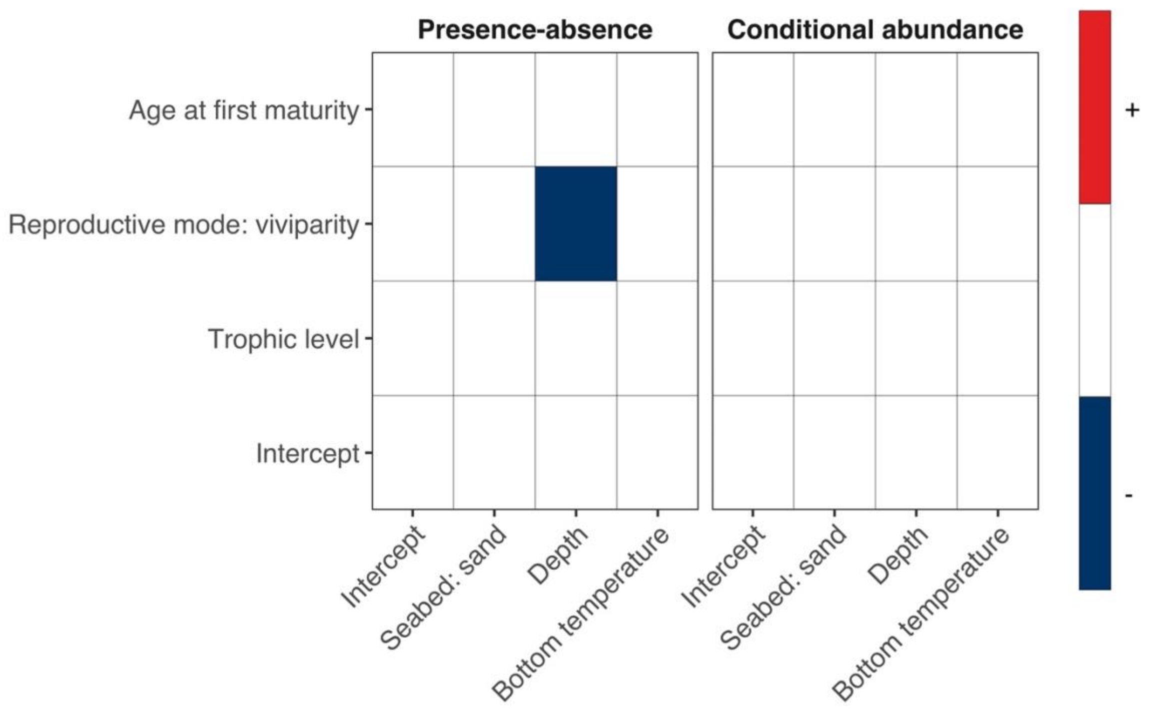

Figure S5. Heatmap of the estimated γ parameters (regression slopes) which link species traits to species niches. The left panel represents the presence-absence (PA) model while the right panel the conditional abundance (ABU) model. Blue color indicates parameters that are estimated to be negative with at least 95% posterior probability, while responses that did not gain strong statistical support are shown in white. No parameter is estimated to be positive with at least 95% posterior probability.

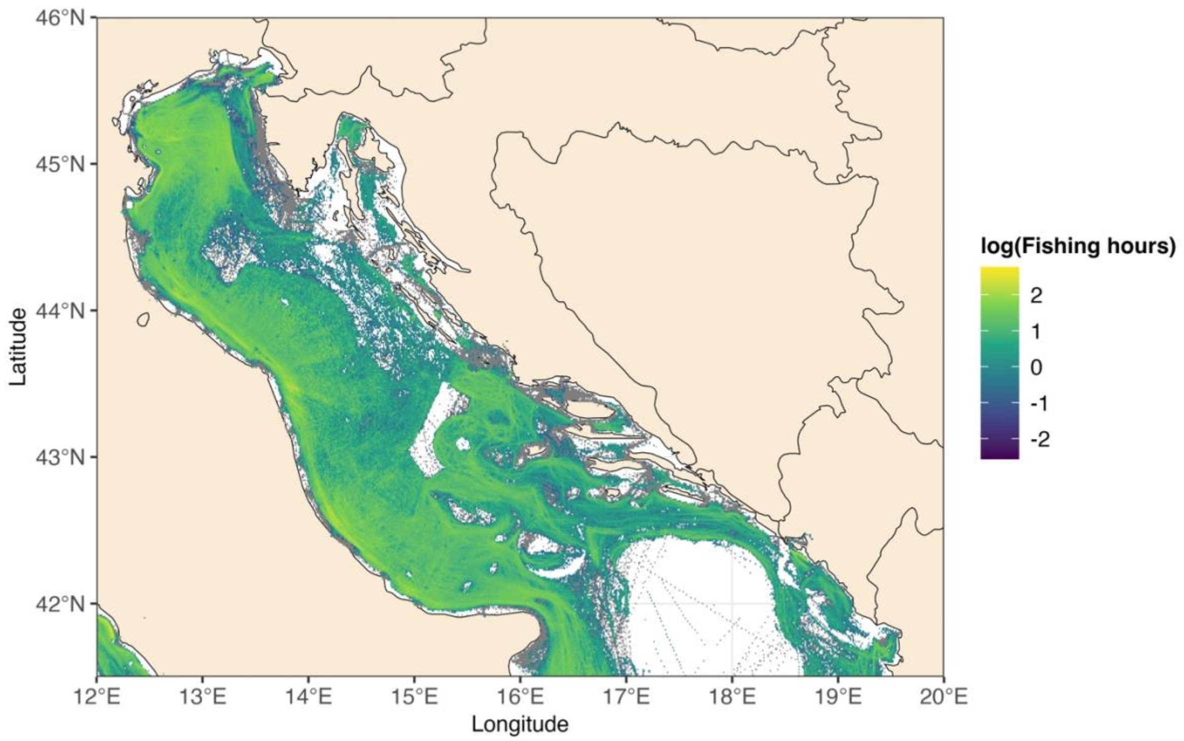

Figure S6. Map showing the estimated spatial distribution of fishing effort in the North-Central Adriatic Sea during the year 2019. Data are obtained from the Automatic Identification System (AIS) and analyzed using Global Fishing Watch (https://globalfishingwatch.org/). The map specifically shows trawlers as the gear type used. Fishing effort is expressed in fishing hours.

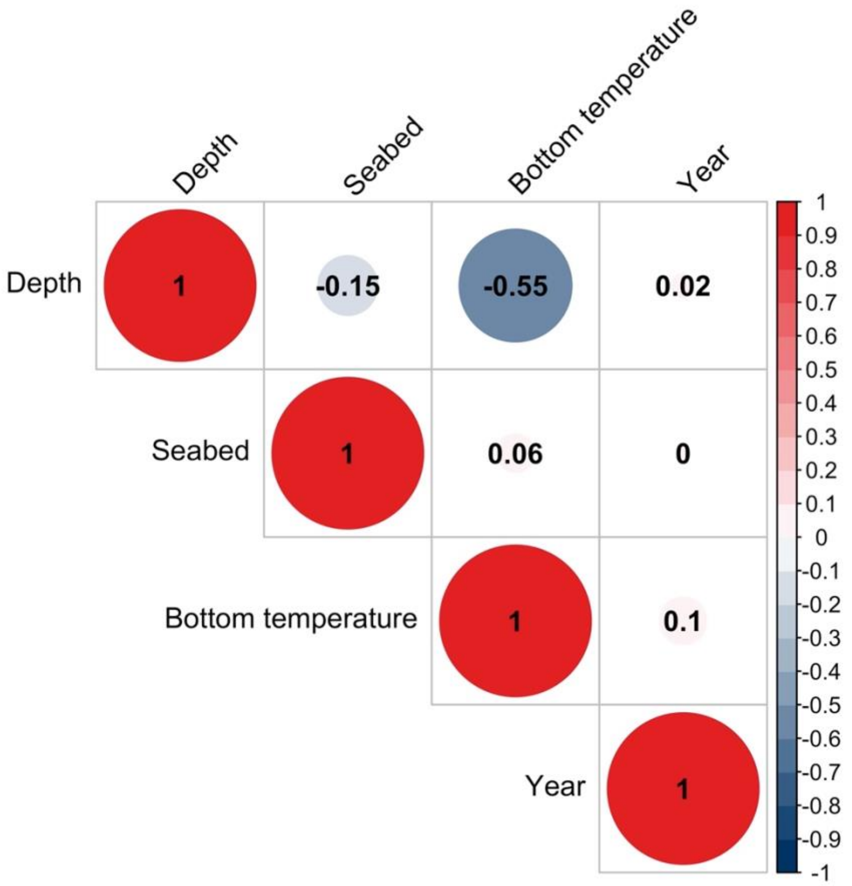

Figure S7. Pearson pairwise correlation coefficients for the environmental covariates included in the analysis. Red (blue) colour indicates positive (negative) coefficients, which are reported inside the circles.

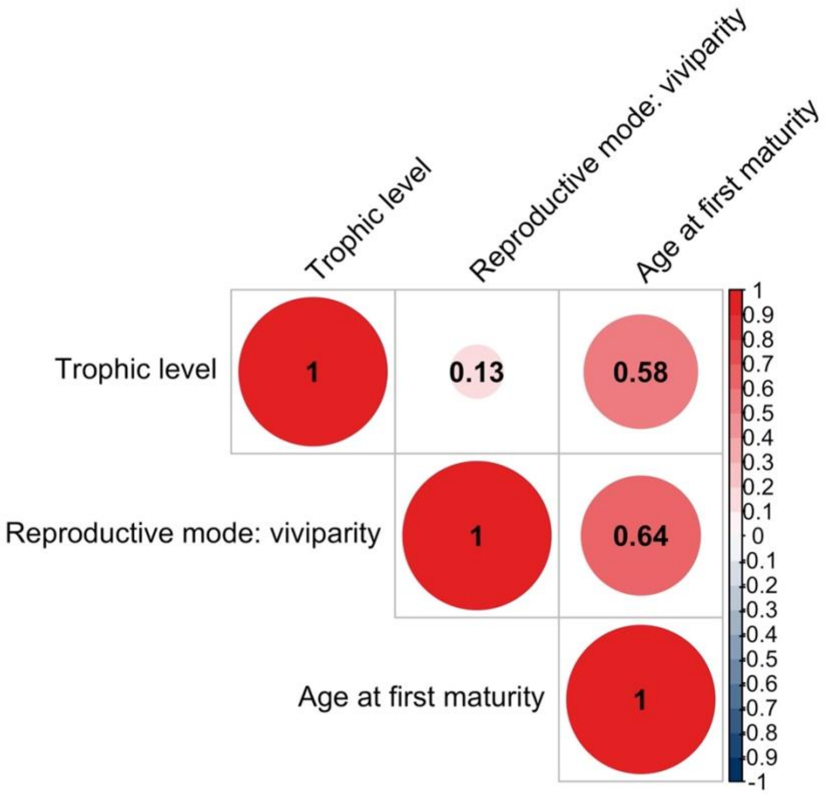

Figure S8. Pearson pairwise correlation coefficients for the traits included in the analysis. Red colour indicates positive coefficients, which are reported inside the circles.

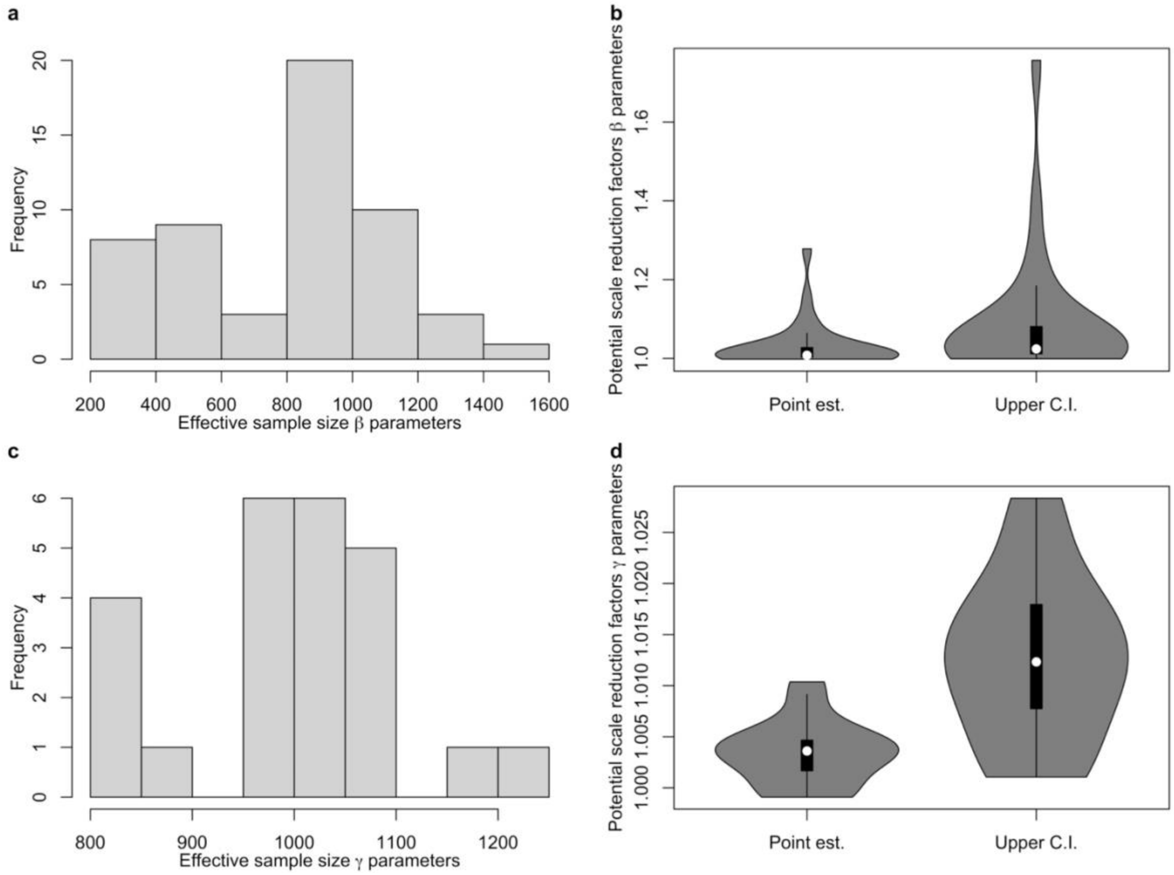

Figure S9. Model diagnostics for the presence-absence (PA) model. The panels correspond (a-b) to the β parameters and (c-d) to the γ parameters. The panels on the left measure MCMC in terms of the effective sample size and the panels on the right in terms of the potential scale reduction factor.

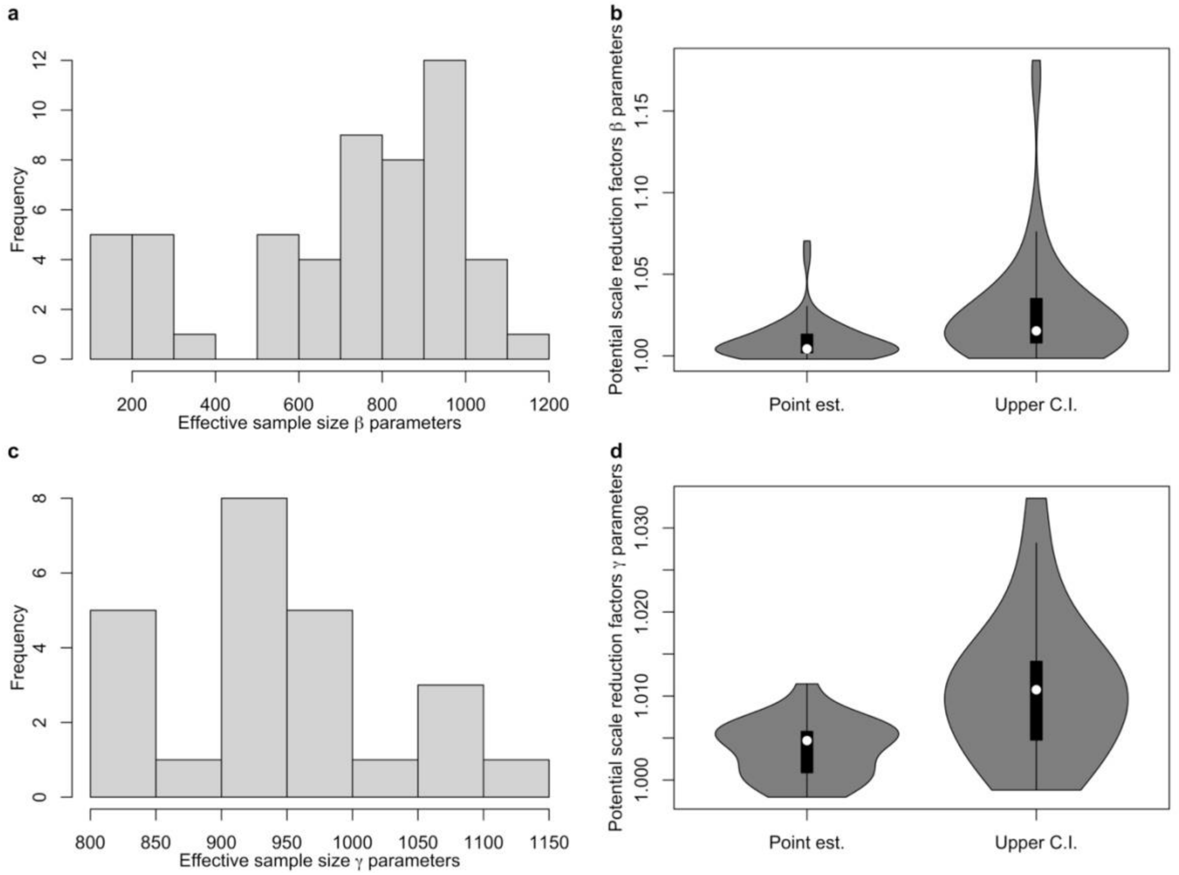

Figure S10. Model diagnostics for the conditional abundance (ABU) model. The panels correspond (a-b) to the β parameters and (c-d) to the γ parameters. The panels on the left measure MCMC in terms of the effective sample size and the panels on the right in terms of the potential scale reduction factor.

Models' equations

We modeled the occurrence and abundance of each species (denoted as j) in each sampling unit (denoted as i) using a generalised linear model

$$y_{ij} \sim D\left(L_{ij}, \sigma_j^2\right) \qquad (1)$$

where D is the statistical distribution (i.e., probit for the presence-absence model and normal for the conditional abundance), L_{ij} is the linear predictor, and σ_j^2 is the variance term (which is excluded for the probit model).

The linear predictor L_{ij} is modeled as the sum of fixed and random effects

$$L_{ij} = L_{ij}^F + L_{ij}^R \qquad (2)$$

The fixed effects were modeled as a regression

$$L_{ij}^F = \sum_k x_{ik}\,\beta_{jk} \qquad (3)$$

Here, x_{ik} represents the covariate k measured at site i, and β_{jk} represents the response of species j to covariate k, where $x_{i1} = 1$ denotes the intercept.

The species' response to covariates is assumed to follow a multivariate normal distribution

$$\beta_{..} \sim N(\mu_{..}, V \otimes [\rho C + (1-\rho)I]) \qquad (4)$$

Here, $\beta_{..}$ denotes the vector of regression coefficients for all species' response to the covariates, which can be interpreted as species environmental niches. The symbol $\otimes$ denotes the Kronecker product, and $0 \leq \rho \leq 1$ measures the strength of the phylogenetic signal.

The expected niche μ_j models the influence of species-specific traits on species' responses, with

$$\mu_{jk} = \sum_l t_{jl}\,\gamma_{lk} \qquad (5)$$

where t_{jl} is the value of trait l for species j (with $t_{j1} = 1$ denoting the intercept), and γ_{lk} measures the effect of trait l on the response to covariate k. In this equation, C denotes the phylogenetic covariance matrix, and I denotes the identity matrix. When $\rho = 0$, the residual variance is independent among the species, implying that closely related species do not have more similar environmental niches than distantly related ones. When ρ approaches $\rho = 1$, species' environmental niches are fully structured by their phylogeny, with related species having more similar niches than expected by chance, implying niche conservatism.

The random effects are modeled as

$$L_{ij}^R = \sum_{r=1}^{n_f} L_{ij}^{r,R} \qquad (6)$$

where $L_{ij}^{r,R}$ is the linear predictor related to the random effect r. In this case, $r = 1$ represents the random effect of haul, and $r = 2$ represents the random effect of year.

We define “units” as the sampling units at the haul and year level and denote the unit behind sampling unit i as $u^r(i)$. The random effect number r is then defined as

$$L_{ij}^{r,R} = \sum_{h=1}^{n_f^r} \eta_{u^r(i)h}\, \lambda_{hj}^r \qquad (7)$$

Here, the summation goes over n_f^r, which are the number of factors included for random effect r. The $\eta_{u^r(i)h}$ are the “unit loadings,” and the λ_{hj}^r are the “species loadings”. The unit loadings $\eta_{u^r(i)h}$ have an exponentially decaying correlation structure, where

$$\eta_{.h}^r \sim N(0, \Sigma) \qquad (8)$$

and

$$\sum_{u_1^r u_2^r} = exp\left(-d_{u_1^r u_2^r}/\alpha_h^r\right) \qquad (9)$$

Here, $d_{u_1^r u_2^r}$ is the distance in space or time between the units u_1^r and u_2^r, and α_h^r is the spatial or temporal scale associated with the factor number h of the random effect r.
